# Using reflective poems to describe the lived experiences of street children and adolescents in Ghana

**DOI:** 10.1080/02673843.2014.978342

**Published:** 2014-11-07

**Authors:** Kwaku Oppong Asante, Anna Meyer-Weitz

**Affiliations:** ^a^ Discipline of Psychology, School of Applied Human Sciences, University of KwaZulu-Natal, King George V Avenue, Howard College Campus, Durban4041, South Africa

**Keywords:** homeless youth, resilience, health risk behaviour, qualitative research

## Abstract

These two poems emerged from my qualitative research with homeless youth in Accra Central, Ghana. I was overwhelmed at how this method of research rarely used in Ghana offers a researcher the opportunity to capture participants' subjective feelings, and the complexities of their perceptions and experiences of a phenomenon. The aim of the study was to examine the lived experiences of street youth and to explore factors that enhance their survival on the street. These reflective poems shed light on the experiences of both the street youth and researcher, as captured in my reflective journal during the research. It was difficult winning the trust of the street youth, but when the trust was won, it became a worthy journey to understanding the complexities of their daily lives.

## Introduction

Having done both undergraduate and master's degrees using quantitative approaches to research and the fact that it was the dominant and popular paradigm in Ghana, the researcher used a qualitative approach to examine the lived experiences of homeless youth in Ghana as part the researcher's doctoral study. This methodological approach allowed the researcher to examine and describe the subjective experiences of the participants in detail using their own words and actions (Silverman, 2013). This approach to research also help the researcher to capture how those being interviewed view their world, to learn their terminology and judgments, and to capture the complexities of their perceptions and experiences (Neuman, 2011; Ungar, 2004).

This study, with the aim of exploring the lived experiences of street youth and to find out the factors that enhance their survival on the street, was conducted in 2012 using a purposively selected sample of 16 homeless adolescents (9 males and 7 females) from the Central Business District, Accra, Ghana. A semi-structured interview schedule consisting of questions on issues ranging from reasons and the circumstances which led participants to leave home, how they cope and survive and how living on the street has influenced their lifestyles and behaviours, was used to conduct interviews in a preferred language and at a place of convenience to the participant. Conventional ethical processes were followed. Institutional ethical clearance to conduct this study was obtained from the Human and Social Sciences Ethics Committee of the University of KwaZulu-Natal, South Africa (Protocol number: HSS/1144/012D) and the Department of Social Welfare, Accra, Ghana. Participation in the study was voluntary and the homeless youth were informed that they could withdraw consent at any time during the interview process.

With the help of a reflective journal which was kept during the data collection process, the common themes identified from my study and the researchers own experiences were summarised in a form of two descriptive poems. These poems entitled *Why choose the streets?* and *Narratives of street life* are described in the following sections. The former presents the researcher's journey to finding the participants as well as the various reasons given by the participants for their state of homelessness. The latter, reflects on the life as lived on the street by the participants.

### Why choose the streets?

Walking bare footed, in tattered clothes

From railways stations to bus stations

From big markets to slums

On pavements, to loggia's

In a quest to find them, I searched

Through a hustling and bustling city center

Visible, yet apprehensive

I mean no harm, yet they were not convinced

Just when I thought of giving up on them

They consented to interaction

Stories were told

Painfully and shamefully

Of how they ended up on the street

“Abusive mamas”

“Sexually exploitative papas”

“Fleeing from home to be free”

As the pains were no longer to be contained

Escaping from poverty bosom to blossom

Now, lonely walking the streets of the unknown

The new way as it's known.

### Narratives of street life

“We do smoke ‘weed’, it makes us feel good”

“You become weak, if you don't have it”

“When I am lonely, I smoke it more”

“When I am with my friends, they give me more”

“I can't help it, than to continue with it”

With friends you are happy in different ways

Without them, you go hungry for days

If you don't give me when you have it

I will not give you when I get it

It's a tit for tat situation

No pain, no gain

We sleep in a group

To avoid being raped

If you sleep too deep; your money gets feet

If you sleep alone, you are sexual meat

Yet, silence is told and behold

Or either weakness will show

Here on the street; that's not to be known

“We are not lazy”; maybe a bit crazy

Working makes us busy

Scraps are our route to making money

Playing football is our way to nakeding our minds

Music and dance are our style to barring our miseries

Do you want to hear more?

“Religion is our core”

And “Jah lives forevermore”

So we are restored

## Conclusion

It was a hard task gaining the confidence of the street youth for the interview, but once this had been done, they seemed to use the opportunity to narrate their stories of how they ended up on the street, and what their experiences of being homeless have been like. Many of them narrated various reasons that led them to the street ranging from extreme family poverty to the quest for freedom from parental control. Some of them were overwhelmed by the harsh conditions on the street but others saw these as normal challenges of streetism.

The experiences of females were remarkably different from their male counterparts as they are exposed to severe health risk behaviours including sexual violence, abuse and possible rape. Notwithstanding these problems encountered on the street, I was surprised by how participants derive their source of strength to navigate the odds of the streets. This, they do by deriving meaning from their environment through activities such as listening to music and playing football, strong religious beliefs, and good and reciprocal friendships. The reflective journal which I kept during the data-collection process helped me capture their moods; something that my voice recorder could not completely do.
